# *In vivo* techniques for assessment of insulin sensitivity and glucose metabolism

**DOI:** 10.1530/JOE-23-0308

**Published:** 2024-01-31

**Authors:** Margaret K Hahn, Adria Giacca, Sandra Pereira

**Affiliations:** 1 Centre for Addiction and Mental Health, Toronto, Ontario, Canada; 2 Institute of Medical Sciences, University of Toronto, Toronto, Ontario, Canada; 3 Department of Pharmacology, University of Toronto, Toronto, Ontario, Canada; 4 Department of Psychiatry, University of Toronto, Toronto, Ontario, Canada; 5 Banting & Best Diabetes Centre, Toronto, Ontario, Canada; 6 Department of Physiology, University of Toronto, Toronto, Ontario, Canada

**Keywords:** whole animal physiology, glucose metabolism, insulin sensitivity, glucose tolerance

## Abstract

Metabolic tests are vital to determine *in vivo* insulin sensitivity and glucose metabolism in preclinical models, usually rodents. Such tests include glucose tolerance tests, insulin tolerance tests, and glucose clamps. Although these tests are not standardized, there are general guidelines for their completion and analysis that are constantly being refined. In this review, we describe metabolic tests in rodents as well as factors to consider when designing and performing these tests.

## Introduction

Breakthroughs in metabolic research rely upon *in vivo* studies using animal models, usually rodents. Assessment of glucose metabolism in rodents is a key component of diabetes research. Although general guidelines for quantification and interpretation of glucose metabolism experiments exist, such guidelines are constantly evolving. In this review, we describe the most common *in vivo* techniques currently used to assess glucose metabolism in rats and mice as well as factors to consider when utilizing such techniques. The purpose of this review is two-fold: (i) to highlight recent developments in performing as well as interpreting results of metabolic tests in rodents and (ii) to provide an easy-to-follow introduction to glucose metabolism methodology in rodents from theoretical and practical perspectives. We discuss metabolic techniques in both rats and mice because both species are commonly used in metabolic research. We compare, as applicable, metabolic protocols used in rodents with those used in humans to understand the translatability of metabolic tests.

## Determining insulin sensitivity and glucose metabolism *in vivo*

### Glucose tolerance test

The glucose tolerance test (GTT) assesses the response (i.e. circulating glucose concentration) to a glucose load (American Diabetes Association 2014). Circulating glucose concentrations during a GTT are the net result of two crucial factors: insulin secretion by pancreatic β cells and insulin sensitivity (Ferrannini & Mari 2014). The relationship between insulin secretion rate and insulin sensitivity is hyperbolic and their product is the disposition index (Ferrannini & Mari 2014). To determine the role of insulin secretion and insulin sensitivity in GTT results, additional tests are required to assess each of these factors. Insulin sensitivity refers to the magnitude of insulin’s metabolic effects, including insulin-stimulated suppression of glucose production by the liver and insulin-stimulated glucose uptake by skeletal muscle (Pereira & Giacca 2011). Therefore, mouse A is more insulin sensitive than mouse B if the metabolic effects of insulin, at a given insulin concentration, are greater in mouse A. Plasma insulin concentrations during the GTT are commonly used as markers of insulin secretion (Pereira *et al.* 2019, Huynh *et al.* 2010), but it is important to note that plasma insulin concentrations in peripheral blood vessels (e.g. carotid artery, saphenous vein, tail vein) are the net result of insulin secretion and insulin clearance (Najjar *et al.* 2023). The gold standard technique for *in vivo* assessment of glucose-stimulated insulin secretion (i.e. β cell function) is the hyperglycemic clamp (Pereira & Giacca 2011). Another less studied variable that affects glucose tolerance is glucose effectiveness, which is the ability of glucose to inhibit its own production and stimulate its own uptake (Best *et al.* 1996, Lam *et al.* 2005, Ayala *et al.* 2010, Carey *et al.* 2020). Glucose effectiveness is calculated using (i) the frequently sampled intravenous glucose tolerance test (IVGTT) with minimal model analysis or (ii) the pancreatic hyperglycemic clamp to determine the effect of selectively elevating glucose concentrations on endogenous glucose production (EGP) and glucose uptake (Tokuyama & Suzuki 1998, Ahrén & Pacini 2002, Henderson *et al.* 2005, Schwartz *et al.* 2013, Karstoft *et al.* 2017, Carey *et al.* 2020). It appears that glucose effectiveness is a more important factor in determining circulating glucose concentrations during an OGTT in mice vs humans (Bruce *et al.* 2021).

The route of administration of the glucose load can be oral (usually more specifically directly into the stomach via gavage), intravenous (into blood vessel), or intraperitoneal (into peritoneal cavity), leading to OGTTs, IVGTTs, and IPGTTs, respectively (Turner *et al.* 2011). The latter is not used in humans. OGTT is the most physiological of the GTTs because it involves ingestion of glucose into the lumen of the gastrointestinal tract, which is the usual route through which glucose enters the body (Ferrannini & Mari 2014). Glucose administered orally is absorbed in the gastrointestinal tract and enters the portal circulation; therefore, the first organ that glucose will reach is the liver. Glucose administered intraperitoneally will also enter the portal vein because its main fate is being absorbed into mesenteric blood vessels (Turner *et al.* 2011). In contrast, if glucose is delivered intravenously (e.g. via the jugular vein (Frangioudakis *et al.* 2008)), the liver is not the first organ to be encountered by glucose.

Results for OGTT, IVGTT, and IPGTT may be different because the underlying physiological mechanisms are unique. When glucose that is consumed orally, either by gavage or starting at the mouth, reaches the intestinal lumen, it is sensed by enteroendocrine K and L cells, which will release the incretin hormones glucose-dependent insulinotropic polypeptide (GIP) and glucagon-like peptide 1 (GLP-1), respectively (Campbell & Drucker 2013, Reimann & Gribble 2016). Incretin hormones act on pancreatic β cells to potentiate insulin secretion (Campbell & Drucker 2013). This ‘incretin effect’ is not observed in IVGTTs and IPGTTs (Ayala *et al.* 2010, Ferrannini & Mari 2014, Alquier & Poitout 2018). Furthermore, the presence of glucose in the mouth per se increases insulin secretion through a neural pathway (i.e. cephalic-phase insulin release), which in turn improves glucose tolerance (Glendinning *et al.* 2015). In healthy rodents of both sexes, at a given dose of glucose, lower blood glucose levels are obtained in OGTTs vs IPGTTs (Benedé-Ubieto *et al.* 2020, Small *et al.* 2022). This is associated with greater circulating insulin concentrations and inhibition of glucose production in OGTTs vs IPGTTs (Small *et al.* 2022). The fact that lower blood glucose levels are achieved by OGTT may also be caused by slow glucose absorption from the gastrointestinal tract (Small *et al.* 2022). Glucose effectiveness may be more important in explaining the glucose excursion profile during an IPGTT compared to an OGTT because (i) a robust increase in circulating insulin is lacking during an IPGTT and (ii) glucose appearance kinetics depend on the route of glucose administration, with glucose appearance in the circulation being greater during an IPGTT (Small *et al.* 2022).

Analyses of GTT results consist of comparing (i) blood glucose concentrations at specific timepoints, (ii) plasma insulin concentrations at specific timepoints (if measured), and (iii) area under the curve for the blood glucose concentration vs time graph for the entire procedure. GTTs typically last 2 h but can be shortened to 1–1.5 h (Ayala *et al.* 2010, Yue *et al.* 2016, Virtue & Vidal-Puig 2021, Small *et al.* 2022). When comparing blood glucose or plasma insulin concentrations between experimental groups throughout the GTT, one should use a two-way ANOVA followed by appropriate *post hoc* tests to minimize type 1 error (i.e. finding a difference between experimental groups where there is none). When the baseline (fasting) blood glucose concentration is different between experimental groups, the percentage change of blood glucose or plasma insulin concentration at each timepoint relative to baseline concentration should not be used and the area under the baseline should be subtracted when calculating area under the curve (Alquier & Poitout 2018, Virtue & Vidal-Puig 2021). Typical glucose doses for GTTs have been summarized elsewhere (Alquier & Poitout 2018).

### Insulin tolerance test

The insulin tolerance test (ITT) is typically used to assess *in vivo* insulin sensitivity in rodents because it is not technically difficult. In humans, the index of insulin sensitivity obtained from the ITT correlates well with whole-body insulin sensitivity quantified with the hyperinsulinemic euglycemic clamp, which is the gold standard technique to assess insulin sensitivity *in vivo* (Pereira & Giacca 2011). To the best of our knowledge, however, these two tests have still not been directly compared in mice (Pereira & Giacca 2011). In the ITT, insulin is usually injected intraperitoneally (i.p.) and circulating glucose concentrations are monitored over 1–2 h (Ayala *et al.* 2010, Pereira *et al.* 2019, Virtue & Vidal-Puig 2021). When comparing two groups of mice, one of the groups should have a maximal decrease in blood glucose concentration of ~50% so that differences between groups, if present, can be detected (Ozcan *et al.* 2006, Huynh *et al.* 2010, Engström Ruud *et al.* 2020). Interpretation of ITT results depends on the extent of blood glucose concentration drop in response to insulin; the greater the decrease in blood glucose concentration, the more insulin sensitive the animal is. However, blood glucose concentrations should not drop excessively because this will trigger a counterregulatory response, which will confound the ITT results; the threshold for initiation of the counterregulatory response is considered to be ~4mM, but it is specific to a given mouse population (Ayala *et al.* 2010, Alquier & Poitout 2018, Virtue & Vidal-Puig 2021). Moreover, it is the first 20–30 min of the ITT that are important to determine insulin sensitivity because the half-life of insulin is <10 min (Ayala *et al.* 2010, Alquier & Poitout 2018, Virtue & Vidal-Puig 2021).

ITT results represent insulin sensitivity at the level of the whole body (Ayala *et al.* 2010). The approach and caveats to analysis of ITT results are similar to those for GTT results, as described in the Glucose tolerance test section, except that area above the curve is preferred over area under the curve (Bruin *et al.* 2015, Carper *et al.* 2020, Virtue & Vidal-Puig 2021). In line with the importance of the first 20–30 min of the ITT, the slope of reduction in blood glucose concentrations is described as the best index of insulin sensitivity that can be derived from an ITT (Pereira & Giacca 2011, Alquier & Poitout 2018). However, calculation of this slope is not commonly done in rodents. Similarly, the circulating insulin concentrations achieved after the injection of insulin in an ITT are rarely measured. Nevertheless, this is an important parameter and can become a confounding variable if, despite the same insulin dose, different levels of circulating insulin are achieved (Ahrén & Pacini 2006). High-fat diet (HFD)-fed mice, which are insulin resistant, have decreased insulin clearance and elevated fasting circulating insulin concentrations (Al-Share *et al.* 2015, Kurauti *et al.* 2016). Following administration of insulin during an ITT, circulating insulin concentrations have been reported to be higher in HFD-fed mice compared to standard chow-fed mice (controls) for at least the first 60 min of the ITT (Kurauti *et al.* 2016). Differences in circulating insulin could prevent detection of sensitivity impairment using the ITT. Common insulin doses for ITTs have been published (Alquier & Poitout 2018).

### Pyruvate tolerance test

The pyruvate tolerance test (PTT) measures the extent to which exogenous pyruvate is converted to glucose by the body. Therefore, the PTT assesses the rate of gluconeogenesis with pyruvate as a starting point, but it does not provide information about gluconeogenesis of nonpyruvate substrates, for example glycerol. Gluconeogenesis together with glycogenolysis makes up endogenous glucose production (EGP).

In a PTT, sodium pyruvate is typically injected i.p. and the experimental flow as well as data analysis is similar to that of an IPGTT (Ferreira *et al.* 2012, Hughey *et al.* 2014). Greater circulating glucose concentrations during a PTT implies higher rates of gluconeogenesis in the body (Hughey *et al.* 2014). At the tissue level, the PTT is expected to involve the liver and kidney, which are the two key sites of gluconeogenesis. Brain pyruvate metabolism may also affect PTT results because pyruvate can cross the blood–brain barrier and pyruvate metabolism in the brain lowers EGP in healthy rodents (Cremer *et al.* 1979, Villalba *et al.* 1994, Lam *et al.* 2005). An injection of pyruvate can result in torpor, which is a state of diminished body temperature and activity, in obese mice (Soto *et al.* 2018). Torpor can also affect glucose metabolism (Rubio *et al.* 2023); hence, torpor can be a confounding variable during PTTs. More sophisticated yet complex techniques for quantification of gluconeogenesis *in vivo* exist (Chevalier *et al.* 2006, Yook *et al.* 2023).

### Homeostasis model assessment insulin resistance and quantitative insulin sensitivity check index

Homeostasis model assessment insulin resistance (HOMA-IR) and quantitative insulin sensitivity check index (QUICKI) are indexes of whole-body insulin sensitivity calculated using fasting insulin and glucose concentrations, as reviewed previously (Pereira & Giacca 2011, Meneses *et al.* 2023). HOMA-IR or HOMA-%S (inverse of HOMA-IR) and QUICKI correlate with whole-body insulin sensitivity assessed with the hyperinsulinemic euglycemic clamp in humans, rats, and mice (Pereira & Giacca 2011). A main criticism of these indexes of insulin sensitivity is that they use equations that were initially generated for humans and that analogous equations should be generated for rodents (Alquier & Poitout 2018).

### Hyperinsulinemic euglycemic clamp

The hyperinsulinemic euglycemic clamp is considered the gold standard technique for assessment of *in vivo* insulin sensitivity in humans, rats, and mice. In rats, blood vessel cannulation surgery (jugular vein for exogenous infusions and carotid artery for blood sampling) is performed, and time (~4 days) is required for recovery before performing the clamp in free-moving rats (Pereira *et al.* 2013). In mice, cannulation of the carotid artery is technically challenging; therefore, the experimental protocol is often altered such that mice are typically restrained during the clamp and blood samples are obtained from the tail (Ayala *et al.* 2010).

**Figure 1:**
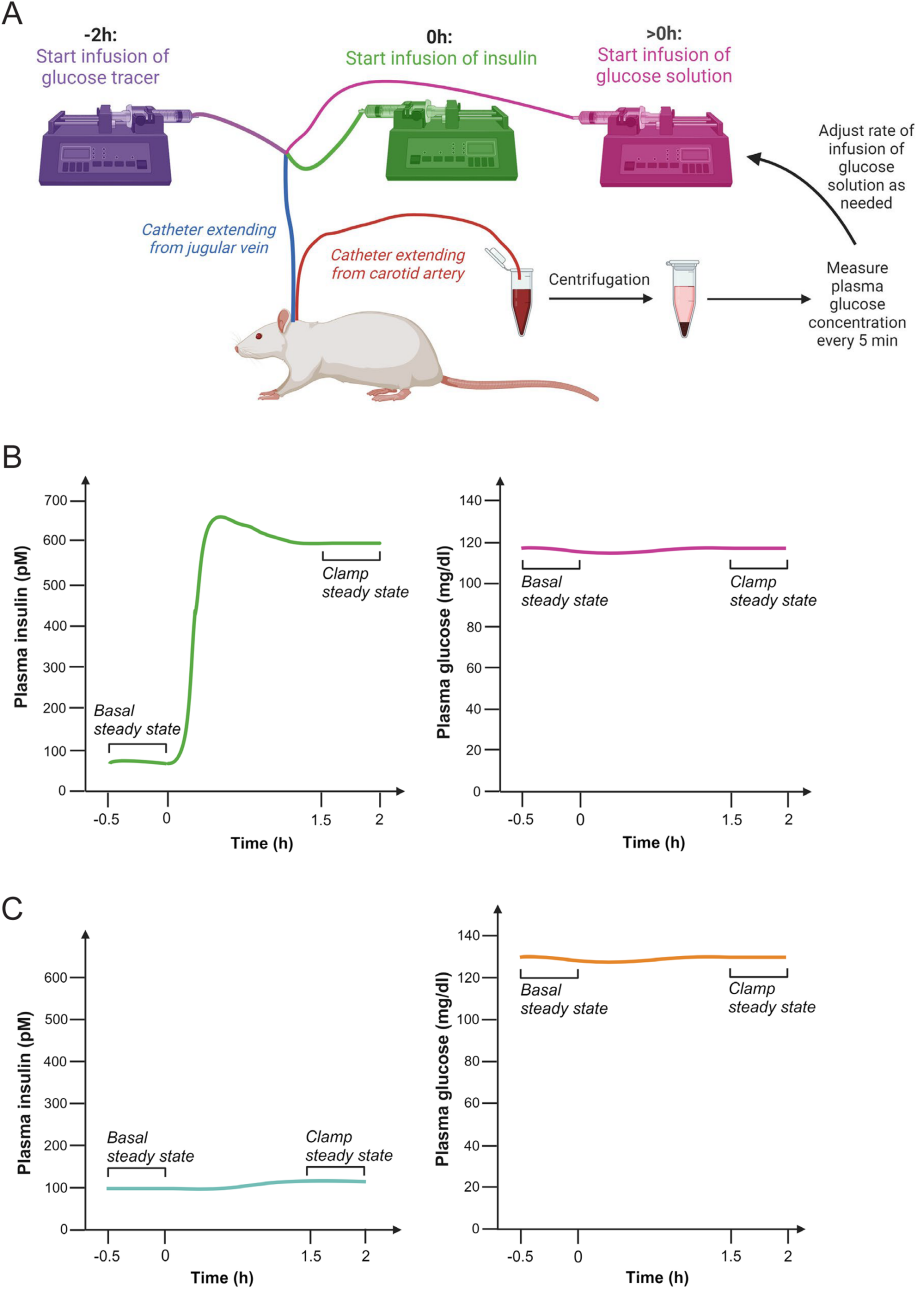
(A) Setup and experimental flow of the hyperinsulinemic euglycemic clamp with tracer methodology in rats. Infusion of a glucose tracer into the jugular vein is initiated 2 h before the start of insulin infusion (i.e. before the start of the hyperinsulinemic euglycemic clamp) and lasts until the end of the clamp. At *t* = 0 h, a constant infusion of insulin into the jugular vein is initiated to increase circulating insulin concentrations (i.e. to obtain hyperinsulinemia) and lasts until the end of the clamp. To achieve euglycemia, which is typically the average plasma glucose concentration for a given rat during the last 30 min before the start of insulin infusion: (i) blood is collected every 5 min from the carotid artery and the plasma glucose concentration is measured and (ii) the rate of infusion of a glucose solution into the jugular vein is adjusted as necessary. The rate of exogenous glucose infusion (Ginf) is lower in rodents with obesity- and type 2 diabetes-associated insulin resistance compared to healthy controls. A hyperinsulinemic euglycemic clamp usually lasts 2 h. (B) Drawing of plasma insulin and glucose concentrations immediately before (basal steady state) and during the hyperinsulinemic euglycemic clamp, including the clamp steady state. Insulin infusion starts at 0 h. Typical insulin and glucose concentrations are also shown. (C) Drawing of plasma insulin and glucose concentrations immediately before (basal steady state) and during the pancreatic euglycemic clamp, including the clamp steady state. Insulin and somatostatin infusions start at 0 h and the pancreatic euglycemic clamp usually lasts 2 h. Typical insulin and glucose concentrations are also shown. Created with BioRender.com.

Experimental protocols for the hyperinsulinemic euglycemic clamp in conscious rats and mice have been published (Ayala *et al.* 2010, Pereira & Giacca 2011, Pereira *et al.* 2013, Hughey *et al.* 2014) and a diagram of the experimental setup for rats is shown in Fig. 1A. The hyperinsulinemic euglycemic clamp involves a constant intravenous (i.v.) infusion of insulin to increase circulating insulin concentrations above baseline, which is typically the insulin concentration after an overnight (rats) or 5–6 h (mice) fast (Ayala *et al.* 2010, Pereira *et al.* 2013). Exogenous glucose solution is then infused i.v. to maintain baseline plasma glucose concentrations (i.e. euglycemia); this requires frequent blood sampling and measuring of plasma glucose (Fig. 1B, which is based on (DeFronzo *et al.* 1979, Pereira *et al.* 2013)). A rat’s own red blood cells are reinfused i.v. throughout the clamp to avoid anemia (Pereira *et al.* 2013). In mice, red blood cells are typically obtained from a ‘donor’ mouse and infused i.v. during the clamp (Ayala *et al.* 2010, Schertzer *et al.* 2011).

Glucose tracer methodology can be combined with the clamp and the glucose kinetics generated may differ depending on the glucose tracer used (Pereira & Giacca 2011). A commonly used tracer is tritiated glucose (3-^3^H-glucose), which allows for quantification of EGP and glucose uptake (glucose utilization) by peripheral tissues such as skeletal muscle. If tracers are not used, then the only parameter of glucose metabolism that is generated is the rate of exogenous glucose infusion (Ginf). Ginf represents whole-body insulin sensitivity, and it is the difference between the rate of glucose utilization and EGP. However, if circulating insulin is sufficiently elevated, EGP will be completely suppressed and glucose utilization will equal Ginf (DeFronzo *et al.* 1983). Two steady states are established in a clamp protocol; one is the baseline/basal steady state, which is the period of time immediately before the start of insulin infusion and the other is the clamp steady state, which is the period of time toward the end of the clamp, typically the last 30 min of a 2-h clamp. A steady state refers to stable concentrations of glucose, insulin, and if tracers are being used, specific activity. Modified versions of the hyperinsulinemic euglycemic clamp also exist. To study protein metabolism, the hyperinsulinemic euglycemic isoaminoacidemic clamp was devised; it differs from the hyperinsulinemic euglycemic clamp in that baseline concentrations of circulating amino acids are maintained throughout the clamp via an exogenous infusion of an amino acid solution (Pereira *et al.* 2008).

In healthy humans and rodents during the hyperinsulinemic euglycemic clamp, inhibition of EGP is accompanied by a robust decrease in plasma concentrations of glycerol and free fatty acids, which is due to inhibition of adipose tissue lipolysis by insulin (Boden *et al.* 1994, Stumvoll *et al.* 2001, Perry *et al.* 2015). In the liver, glycerol is used as a gluconeogenic substrate, while free fatty acids are converted to acetyl CoA, which is an allosteric activator of the gluconeogenic enzyme pyruvate carboxylase (Perry *et al.* 2015). In healthy overnight fasted rats, preventing this decrease in plasma concentrations of glycerol and hepatic acetyl CoA by infusing acetate and glycerol blocks suppression of EGP during the hyperinsulinemic euglycemic clamp (Perry *et al.* 2015). Furthermore, obesity in humans and HFD feeding in rodents elevate plasma free fatty acid concentrations during the hyperinsulinemic euglycemic clamp (Basu *et al.* 2005, Perry *et al.* 2015). Multiple studies have demonstrated that increased plasma free fatty acid concentrations cause hepatic insulin resistance (e.g. Boden *et al.* (1994) and Park *et al.* (2007)). Circulating concentrations of glycerol and free fatty acids increase during fasting in healthy rodents in unclamped conditions (Palou *et al.* 1981, Geisler *et al.* 2016). EGP has been found to remain similar (Heijboer *et al.* 2005, Mutel *et al.* 2011) or become lower (Nunes & Jones 2009) when comparing shorter vs longer (up to 24 h) fasting times in healthy rodents. These findings suggest that elevated circulating free fatty acids and glycerol may not be sufficient to sustain EGP as fasting continues because of other factors, such as depleted glycogen stores (Burgess *et al.* 2005, Nunes & Jones 2009).

Interpretation of glucose kinetics results from the clamp requires measurement of circulating insulin concentrations during the basal and clamp steady states. In the simplest scenario, insulin concentrations at each steady state are similar across experimental groups. If not, then glucose kinetics results have to be divided by insulin concentrations or the alteration in insulin concentrations (Pereira & Giacca 2011). Alternatively, the insulin infusion rate can be altered in one of the groups in order to match the clamp insulin concentrations across groups (Pereira *et al.* 2014). If two experimental groups have different plasma glucose concentrations, the following approaches have been used: (i) divide glucose kinetics results by plasma glucose concentrations or (ii) make the average plasma glucose concentration of the control group the target plasma glucose concentration for all groups during the clamp (Pereira *et al.* 2008, Pereira & Giacca 2011).

In addition to being technically challenging, the clamp is laborious, expensive (especially if tracer methodology is used), and usually terminal. However, it is a powerful technique in metabolic research. Important clamp-specific factors to address in the design, performance, and reporting of this technique have been published recently (Ayala *et al.* 2022).

### Pancreatic euglycemic clamp

The pancreatic euglycemic clamp is used when an investigator wants to test the effect of a treatment without the confounding effect of alterations in endogenous insulin secretion. The pancreatic euglycemic clamp has also been extensively used to study how hormones and nutrients in the brain affect peripheral glucose metabolism; in such studies intracerebroventricular cannulation surgery is performed before vessel cannulation surgery (Lam *et al.* 2005, Castellani *et al.* 2022).

The experimental flow and many aspects of the pancreatic euglycemic clamp are similar to those of the hyperinsulinemic euglycemic clamp (Lam *et al.* 2005, Castellani *et al.* 2022). Similar to the hyperinsulinemic euglycemic clamp, the pancreatic euglycemic clamp requires chronic blood vessel cannulation. Moreover, the pancreatic euglycemic clamp also has two steady states (basal and clamp), usually lasts 2 h, and can be combined with tracer methodology. During the pancreatic euglycemic clamp, however, somatostatin is infused to inhibit endogenous insulin and glucagon secretion by the pancreas and exogenous insulin is infused at rate so that basal insulin concentrations can be achieved during the clamp steady state. Plasma glucose is measured throughout the clamp, and the rate of infusion of a glucose solution (Ginf) is altered as needed to achieve euglycemia (Fig. 1C) (Lam *et al.* 2005, Kowalchuk *et al.* 2017, Castellani *et al.* 2022).

## Factors to consider when assessing glucose metabolism *in vivo*

### Dynamic vs steady state

Techniques that require steady states, like the hyperinsulinemic or pancreatic euglycemic clamp, are essentially reductionist approaches to assess glucose metabolism (Meneses *et al.* 2023). HOMA-IR and QUICKI also assume a steady state during fasting. In contrast, GTTs, ITTs, and PTTs are dynamic tests because, in addition to the main variable, namely, circulating glucose concentration, other variables such as circulating insulin may be changing. Another factor to consider is when, in the feed–fast cycle, the metabolic tests are performed. Clamps, GTTs, ITTs, and PTTs, are usually done in the fasting state to minimize the confounding effect of changes in nutrients and hormones associated with feeding. Moreover, circulating levels of nutrients and hormones as well as glucose metabolism have a circadian rhythm (Ando *et al.* 2016, Stenvers *et al.* 2019). Therefore, the time of day when metabolic tests are performed should be consistent in a given experiment and reported.

### Blood sampling

All *in vivo* glucose metabolism techniques described in this review require blood sampling. When obtaining a blood sample, ideally the rodent should be free-moving and under minimal stress, especially from handling and restraint. Chronic cannulation of blood vessels, such as jugular vein and carotid artery, in rodents allow blood sampling to occur largely under such conditions (Ayala *et al.* 2010). The disadvantage of chronic vessel cannulation is that rodents cannot be kept for long periods of time. In contrast, GTTs, ITTs, and PTTs can be done in longitudinal studies of rodents that do not have vessel cannulation, where a given rodent can be studied multiple times throughout its life as long as blood volume limitations are respected. Therefore, approaches other than chronic vessel cannulation are used to obtain blood in conscious rodents. When only blood glucose is measured, a prick/nick in the tail performed with a needle is generally enough and does not usually require restraint. If larger amounts of blood are needed for other measurements, two common sources of blood are used: the saphenous vein and the tail via tail clipping. Obtaining blood from the saphenous vein involves rapid restraint and can be performed repeatedly during a test (Abatan *et al.* 2008, Pereira *et al.* 2019). When tail clipping is used, restraint may not be necessary if the tail is briefly held (Abatan *et al.* 2008, Moore *et al.* 2017). The extent of stress induced by sampling from the saphenous vein or via tail clip is approximately the same (Abatan *et al.* 2008). However, tail clip is not recommended when larger blood samples are required, and tail clip commonly causes hemolysis (Christensen *et al.* 2009, Ayala *et al.* 2010).

### Measuring blood glucose concentration

Blood or plasma glucose concentrations are typically measured with glucometers that were designed for humans, which usually use ≤5 μL blood (Ayala *et al.* 2010, Togashi *et al.* 2016). Moreover, glucometers usually have a maximal reading of ~33 mM for blood (Pereira *et al.* 2019). The accuracy of glucometers when measuring glucose in rodent blood has been compared to results obtained with a glucose assay kit or glucose analyzer (Togashi *et al.* 2016, Morley *et al.* 2018). The difference in plasma glucose concentrations between various models of glucometers and a glucose assay kit increases as plasma glucose concentration rises (Togashi *et al.* 2016). Furthermore, the direction of this error changes depending on whether mice have been fasted or not (Togashi *et al.* 2016). Therefore, a glucometer type should be used consistently in a given experiment (Ayala *et al.* 2010, Togashi *et al.* 2016) and its results are usually most reliable when glycemia is not excessively high (Togashi *et al.* 2016, Morley *et al.* 2018). When doing GTTs and PTTs in models of diabetes, plasma glucose concentrations can be determined after completion of the tests using a glucose assay kit (Pereira *et al.* 2021).

For clamps, circulating glucose concentrations must be determined as fast as possible (<5 min), which is the case with both glucometers and rapid glucose analyzers. The HemoCue glucose analyzer, which requires ~5 μL of blood to measure plasma glucose concentration in humans, has been used to measure plasma glucose concentration during clamps in mice (Nahle *et al.* 2021). However, the maximal glucose reading for HemoCue is <33 mM. For clamps in rats, glucose concentrations in 5–10 μL plasma samples can be determined using glucose analyzers such as GM9 from Analox (Castellani *et al.* 2022). The latter also has the advantage of measuring glucose concentrations >33 mM.

Continuous glucose monitoring (CGM) involves invasive surgery, namely, surgical implantation of a telemetry probe in the aorta (Evers *et al.* 2020, Kennard *et al.* 2021, Rubio *et al.* 2023). Assuming surgical expertise and funds exist in a laboratory, CGM is a great way to determine circulating glucose concentrations in rats and mice over weeks without handling them (Evers *et al.* 2020, Kennard *et al.* 2021, Rubio *et al.* 2023).

### Fasting duration

Unless glucose metabolism is being specifically assessed in the feeding and postprandial states, all *in vivo* glucose metabolism tests are done in the fasting (postabsorptive) state to avoid the confounding effect of altered concentrations of hormones and nutrients associated with food consumption. The length of fast depends on the species, rodent model, and metabolic test. Glucose production is the sum of glycogenolysis and gluconeogenesis, and as fasting increases, glycogen stores become depleted and the contribution of gluconeogenesis to glucose production increases (Landau *et al.* 1996, Burgess *et al.* 2005). An overnight fast (~12 h) reduces hepatic glycogen content by ~30% in healthy humans (Iwayama *et al.* 2020), but by ~80% in healthy mice (Carper *et al.* 2020). Overnight fasting, which typically lasts 16 h, is considered stressful in mice. Fasting for 24 h also causes strain-dependent alterations in glycogenolysis, with C57BL/6J mice still demonstrating glycogenolysis (Burgess *et al.* 2005). An important species difference is that while a 24 h fast decreases insulin sensitivity in humans (Salgin *et al.* 2009), a 16–18 h fast increases whole-body and peripheral insulin sensitivity in mice (Heijboer *et al.* 2005, Ayala *et al.* 2006). Thus, shorter fasting times are usually advised in mice also for translatability (Ayala *et al.* 2010).

The ideal length of fasting for GTTs and ITTs is an active area of investigation. Four to six hours of fasting is commonly used and advised (Andrikopoulos *et al.* 2008, Ayala *et al.* 2010, Pereira *et al.* 2019, Erener *et al.* 2021, Virtue & Vidal-Puig 2021). Recently, it was reported that shorter fasting (2 h) is ideal when performing ITTs because hepatic glycogen content is similar to the nonfasted state (Carper *et al.* 2020) and there is less risk of fatal hypoglycemia.

### Genetic background

Genetic background of mice affects metabolic parameters such as insulin sensitivity and counterregulatory response to hypoglycemia (Berglund *et al.* 2008); therefore, it is important to state mouse strains in publications. Furthermore, littermate controls should be used (Drucker 2016, Alquier & Poitout 2018).

### Sex of rodents

Among healthy mice, females are more insulin sensitive and have better glucose tolerance than males (Macotela *et al.* 2009). Similarly, women are more insulin sensitive than men (Tramunt *et al.* 2020). This disparity is associated with sex-specific factors such as differences in circulating levels of estrogen and testosterone (Macotela *et al.* 2009, Yan *et al.* 2019, Tramunt *et al.* 2020). Sex hormones also affect body composition (amount and distribution of fat tissue), which is an important determinant of insulin sensitivity (Elbers *et al.* 1999, Macotela *et al.* 2009, Hocking *et al.* 2013). The enhanced insulin sensitivity associated with being female often deteriorates in insulin-resistant states (Tramunt *et al.* 2020, Pereira *et al.* 2021).

Although a shift is occurring, preclinical research often only uses male rodents (Willingham 2022). From a metabolic perspective, one of the reasons may be a combination of the often-increased probability of finding metabolic disturbances in males and the publication bias toward positive findings (Joober *et al.* 2012, Mauvais-Jarvis *et al.* 2017, Tramunt *et al.* 2020). Nevertheless, to maximize the quality of health care for all, while minimizing its cost, it is important to study both sexes/genders from cellular to rodent and eventually clinical research. Results for males and females should not be pooled (Willingham 2022) and flowcharts to design experiments that examine how sex affects metabolism have been published (Mauvais-Jarvis *et al.* 2017).

Another reason why females are less studied than males is the fact that females have cyclic alterations in circulating gonadal hormones, namely, estrogen and progesterone. The estrous cycle and its phases in female rats and mice are analogous to the menstrual cycle in women (Ajayi & Akhigbe 2020). While the menstrual cycle lasts ~28 days, the estrous cycle is shorter, lasting 4–5 days (Ajayi & Akhigbe 2020, Gutierrez-Castellanos *et al.* 2022). Blood glucose concentrations have been found to change throughout the menstrual cycle (Lin *et al.* 2023). It could be argued that the estrous cycle increases variance of metabolic studies; therefore, data from female rodents should be presented by estrous phase (Della Torre *et al.* 2016). The process for tracking the four phases of the estrous cycle is not complex (Ajayi & Akhigbe 2020). However, the requirement to present metabolic data by estrous phase may depend on the primary parameter being investigated (Mauvais-Jarvis *et al.* 2017). Indeed, there is evidence that in cases where estrous phases are not tracked, but sample size is sufficient, females do not show increased variance in various parameters of glucose metabolism (Berglund *et al.* 2012, Mauvais-Jarvis *et al.* 2017, Pereira *et al.* 2019). This may be associated with synchronized estrous cycles of females in a given cage, which occurs at <5 mice per cage due to minimal stress (Mauvais-Jarvis *et al.* 2017). Ultimately, it is up to each investigator to determine if, in addition to studying females, data should be presented by estrous phase. Key factors underlying this decision include the research question and cost.

Estrous cycle is not the only sex-specific factor that can modulate glucose metabolism and potentially increase variance. For example, aggression, which is associated with stress, is more prevalent in male mice (Lidster *et al.* 2019, Benedé-Ubieto *et al.* 2020). Moreover, the stress hormone corticosterone causes greater insulin resistance in male vs female mice (Kaikaew *et al.* 2019). The housing of female mice for metabolic studies usually involves nonpregnant or nonnursing females and occurs in the absence of males; such conditions favor low levels of aggression in female mice (Newman *et al.* 2019).

### Body composition

Doses for GTTs, ITTs, PTTs, and clamps in rodents are usually expressed per kg of body weight. Glucose kinetics in rodents are also usually expressed per kg of body weight (Berglund *et al.* 2008, Pereira *et al.* 2013). Such approaches are appropriate when comparing experimental groups that have similar body composition, especially the amount of fat and lean (fat-free) mass. If this is not the case, then it has been suggested that the amount of lean mass should be determined and the doses should be normalized to lean mass, not body weight (Alquier & Poitout 2018). The underlying rationale is that glucose uptake is greater in lean tissue than in fat tissue (Alquier & Poitout 2018). However, the superiority of lean body mass over body weight for the hyperinsulinemic euglycemic clamp is being questioned in humans (Ter Horst & Serlie 2020). It appears that this issue will be resolved when the different contributions of adipose tissue during the different metabolic tests are clarified (Ter Horst & Serlie 2020).

### Age

Insulin sensitivity and glucose tolerance decline with age in rodents and humans (Chang & Halter 2003, Benedé-Ubieto *et al.* 2020). Thus, the age of the rodents should be reported and matched across experimental groups. From a clinical perspective, there is a need to study metabolism throughout the life span, including menopause and andropause (Kautzky-Willer *et al.* 2012, Alquier & Poitout 2018).

## Assessment of *in vivo* glucose metabolism in rodent models in obesity and type 2 diabetes research

There are many excellent published papers that use *in vivo* techniques to assess glucose metabolism in rodent models of obesity and type 2 diabetes. We will highlight some of the key findings from three of these papers in the current review. First, hyperinsulinemic euglycemic clamps were utilized to conclude that knocking down pyruvate carboxylase in adipose tissue and liver with antisense oligonucleotides ameliorates hepatic insulin sensitivity in male HFD-fed rats, which are a model of obesity, and male Zucker diabetic fatty rats, which are a model of type 2 diabetes (Kumashiro *et al.* 2013). Second, tamoxifen-inducible adipocyte-specific insulin receptor and insulin growth factor 1 knockout mice were generated and characterized (Sakaguchi *et al.* 2017). Two days following tamoxifen administration, male double knockout mice were hyperglycemic, glucose intolerant based on OGTT results and insulin resistant based on ITT as well as HOMA-IR results. Moreover, HOMA-IR was used to track insulin sensitivity over time, and it was found that insulin sensitivity was similar between male double knockout and control mice by 30 days post tamoxifen administration. Third, male and female mice lacking complement factor 5, which is part of the innate immune system, were placed on an HFD and studied (Winn *et al.* 2023). Using IPGTTs, it was concluded that in the context of HFD-induced obesity, knocking out complement factor 5 only affects (deteriorates) glucose tolerance in male mice. Hence, these examples support the importance of *in vivo* techniques for assessment of glucose metabolism in understanding the mechanisms of obesity, type 2 diabetes, and insulin resistance as well as new treatments for metabolic disorders.

## Conclusion

Methods of *in vivo* tests of glucose metabolism should be detailed for readers to repeat experiments and to understand the context of results. Preclinical research is valuable if it is translatable to humans (Drucker 2016); therefore, one must frequently question and answer how metabolic tests in rodents relate to human physiology. In this review, we have described the commonly used techniques for the assessment of insulin sensitivity and glucose metabolism in rodents. We have also highlighted the pros and cons of each technique. Furthermore, we have discussed key factors that can affect glucose metabolism, such as fasting duration and sex of the rodents. Other factors that may cause stress and alter glucose metabolism have begun to be described in the literature, such as temperature, method of cage change, and even the sex of the scientist (Sorge *et al.* 2014, Drucker 2016, Georgiou *et al.* 2022, Kennard *et al.* 2022). Regarding the latter, stress responses in mice vary depending on the sex of the investigator handling the mice and merely because men and women give off different scents (Sorge *et al.* 2014, Georgiou *et al.* 2022). It will be interesting to determine how additional factors such as these can be utilized to further optimize metabolic tests in rodents in the future.

## Declaration of interest

MKH received consultant fees from Alkermes, Inc.

## Acknowledgement

This paper forms part of a themed collection on ‘Insulin Resistance and Type 2 Diabetes Mellitus’. The guest editors for this collection were Matthias Blüher, Stefan Bornstein, and Martin Haluzík.

## Funding

Grants from the Banting and Best Diabetes Centre (BBDhttp://dx.doi.org/10.13039/100017412C), Canadian Institutes of Health Researchhttp://dx.doi.org/10.13039/501100000024 (CIHR), and PSI Foundation were awarded to MKH. MKH also has support from an Academic Scholars Award from the Department of Psychiatry, University of Torontohttp://dx.doi.org/10.13039/100015070, and holds the Kelly and Michael Meighen Chair in Psychosis Prevention.
